# Prognostic impact of glioblastoma stem cell markers OLIG2 and CCND2

**DOI:** 10.1002/cam4.2592

**Published:** 2019-09-30

**Authors:** Christelle Bouchart, Anne‐Laure Trépant, Matthieu Hein, Dirk Van Gestel, Pieter Demetter

**Affiliations:** ^1^ Department of Radiation‐Oncology Institut Jules Bordet Université Libre de Bruxelles Brussels Belgium; ^2^ Department of Pathology Erasme University Hospital Université Libre de Bruxelles Brussels Belgium; ^3^ Department of Psychiatry and Sleep Laboratory Erasme University Hospital Université Libre de Bruxelles Brussels Belgium; ^4^ Department of Pathology Institut Jules Bordet Université Libre de Bruxelles Brussels Belgium

**Keywords:** cyclin D2, glioblastoma, immunohistochemistry, neoplastic stem cells, OLIG2, radiotherapy

## Abstract

**Aims:**

Glioblastoma (GBM) is the most common and lethal malignant brain tumor in adults. Glioma stem cells (GSCs) are implicated in this poor prognosis and in radio(chemo‐)resistance. We have previously demonstrated that among potentially highly specific GSC markers oligodendrocyte lineage transcription factor 2 (OLIG2) appears to be the most specific and cyclin D2 (CCND2) the only one related to cell cycle regulation. The purpose of this work was to investigate the clinical significance and the evolution of OLIG2 and CCND2 protein expression in GBM.

**Methods and results:**

Immunohistochemical expression analysis of Olig2 and Ccnd2 was carried out on a cohort of human paired GBM samples comparing initial resections with local recurrent tumors after radiation therapy (RT) alone or radio‐chemotherapy with temozolomide (RT‐TMZ). Uni‐ and multivariate logistic regression analysis revealed that significant risk factors predicting early mortality (<12 months) are: subtotal surgery for recurrence, time to recurrence <6 months, Ccnd2 nuclear expression at initial surgery ≥30%, and Olig2 nuclear expression <30% at second surgery after RT alone and RT‐TMZ.

**Conclusions:**

We demonstrated that patients for whom nuclear expression of Olig2 becomes low (<30%) after adjuvant treatments have a significantly shorter time to recurrence and survival reflecting most probably a proneural to mesenchymal transition of the GSCs population. We also highlighted the fact that at initial surgery, high nuclear expression (≥30%) of CCND2, a G1/S regulator specific of GSCs, has a prognostic value and is associated with early mortality (<12 months).

## Graphical abstract

We investigated by immunohistochemistry the clinical significance and the evolution of Oligodendrocyte lineage transcription factor 2 (OLIG2) and cyclin D2 (CCND2) protein expression, two markers highly specific of glioma stem cells, on a cohort of human paired glioblastoma samples comparing initial resections with recurrent tumors after radiation therapy alone or radio‐chemotherapy with temozolomide according to the Stupp regimen. Our study highlighted for the first time the prognostic value of high nuclear expression of CCND2 at initial surgery which is associated with early mortality and patients for whom nuclear expression of OLIG2 becomes low after adjuvant treatments have a significantly shorter time to recurrence and survival reflecting most probably a proneural to mesenchymal transition of the stem cells population.

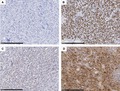

## 1 INTRODUCTION

Glioblastoma (GBM) (World Health Organization [WHO] grade IV glioma) is the most common and lethal malignant brain tumor in adults.^1^ To better define GBM entities, the 2016 WHO classification now integrates to histological features the isocitrate dehydrogenase (IDH) mutation status, one of the most important genetic alterations found in GBM, as the few IDH‐mutant present a more favorable prognosis.^1^ However over the past decades despite some improvements in surgical and radio‐chemotherapeutics treatments and the multiplicity of clinical trials testing new therapies without major success so far,^2^ GBM remains incurable with a median survival of only 12‐18 months^3^ and up to 31 months for IDH‐mutant.^4^ Several factors could contribute to this poor prognosis; the most important appears to be related to the presence of a population of radio/chemoresistant cells with stem‐like properties.^5^, ^6^, ^7^, ^8^ The glioma stem cells (GSCs) subpopulation is capable of self‐renewal, persistent proliferation, dedifferentiation, multipotency and has the ability to be highly tumorigenic allowing for tumor regrowth after standard treatments.^9^ Therefore, developing additional therapeutic strategies targeting and eliminating the GSCs component is crucial to one day render GBM curable. The issue when studying GSCs comes from the fact that they represent a heterogeneous population difficult to identify by routine methods. Today, the gold standard for the determination of GSCs remains the capability of these cells to reshape the complexity of the initial patient tumor after serial orthotopic transplantation assays into mice brains.^8^ Many studies have explored known normal stem cell markers such as CD133 or CD44 to recognize and enrich GSC cultures with flawed results as other cells that do not express such markers also display tumorigenic capacities.^5^, ^10^, ^11^ A universal marker appearing illusory to obtain, a combination of biomarkers seems the best way to explore GSCs for potential routine identification. By performing a comparative analysis of differentially expressed genes between differentiated and GSC enriched cultures from similar DNA chip microarray platforms, we have previously determined a panel of eight genes potentially highly specific of GSCs. Among them, oligodendrocyte lineage transcription factor 2 (OLIG2) appears as the most specific and cyclin D2 (CCND2) as the only one related to cell cycle regulation.^12^ The purpose of this work was to investigate by immunohistochemistry (IHC) the clinical significance and the evolution of OLIG2 and CCND2 protein expression on a cohort of human paired GBM samples comparing initial resections with recurrent tumors after radiation therapy (RT) alone or radio‐chemotherapy with temozolomide (RT‐TMZ) according to the Stupp regimen.^3^

## 2 MATERIALS AND METHODS

### 2.1 Retrospective clinical series

We analyzed the protein product of OLIG2 and CCND2, two gene candidates for GSC biomarkers, in a retrospective paired samples cohort of 72 GBM. Uncommon histopathological subtypes of GBM were not included. The patients underwent an initial subtotal or macroscopically complete tumor resection at the Erasme University Hospital (Brussels, Belgium) between April 1990 and March 2014. The patients then received adjuvant therapy according to the standard guidelines in use at the time of the initial surgery: either RT alone (n = 37) or RT‐TMZ according to the Stupp regimen (n = 35). The Stupp regimen corresponds to fractionated conformal RT (60 Gy in 30 fractions given 5 days per week for 6 weeks) with continuous daily oral TMZ (75 mg/m^2^ of body‐surface area per day, 7 d/wk from the first to the last day of RT) followed by six cycles of adjuvant TMZ (150‐200 mg/m^2^ for five every 28 days).^3^ At recurrence in the same area of the primary tumor, the patients underwent a second subtotal or macroscopically complete tumor resection at the Erasme University Hospital between August 1991 and September 2014. All tissue samples (paraffin blocks) analyzed in this study came from the archives of the Department of Pathology of the Erasme University Hospital. This study was approved by the Erasme University Hospital Ethics Committee (P2014/290). The clinical data recorded for each patient are summarized in Table 1.

**Table 1:** Sample description according to adjuvant treatment received (RT alone or RT‐TMZ)

	Whole cohort	RT alone	RT‐TMZ	*P*‐value
CCND2 nuclear expression (%)	*P* < .001	*P* = .013	*P* = .001	
Before adjuvant treatment	31.00 ± 19.00	32.00 ± 19.00	31.00 ± 20.00	.893^a^
After adjuvant treatment	20.00 ± 18.00	20.00 ± 19.00	19.00 ± 18.00	.731^a^
OLIG2 nuclear expression (%)	*P* = .003	*P* = .027	*P* = .044	
Before adjuvant treatment	43.00 ± 23.00	44.00 ± 22.00	41.00 ± 24.00	.707^a^
After adjuvant treatment	31.00 ± 22.00	32.00 ± 23.00	30.00 ± 22.00	.764^a^
Age at diagnosis	54.23 ± 11.63	54.69 ± 11.22	55.04 ± 11.69	.897^a^
Gender (male)	62.50%	59.50%	65.70%	.584^b^
Number of lesions (multiple)	12.50%	16.22%	8.87%	.327^b^
Preoperative corticosteroids (yes)	80.56%	81.08%	80.00%	.908^b^
MGMT methylation status				NA
Unknown	72.22%	100% (n = 37)	42.85% (n = 15)	
Methylated	11.11%	0%	22.86% (n = 8)	
Not methylated	16.67%	0%	34.29% (n = 12)	
GBM				NA
NOS	72.22%	100% (n = 37)	42.85% (n = 15)	
IDH‐mutant	4.17%	0%	8.57% (n = 3)	
IDH‐wildtype	23.61%	0%	48.57% (n = 17)	
Initial type of surgery (subtotal)	40.28%	37.84%	42.86%	.664^b^
Time to first recurrence (mo)	6.90 (4.54‐10.90)	6.00 (4.20‐8.67)	9.03 (4.83‐16.37)	.018^c^
Type of surgery at recurrence (subtotal)	43.06%	56.76%	57.14%	.974^b^
Time between the two surgeries (mo)	8.28 (5.52‐12.00)	6.93 (5.20‐10.30)	9.60 (7.10‐16.37)	.038^c^
Overall survival (mo)	15.69 (11.63‐23.14)	14.04 (11.28‐20.42)	15.83 (13.10‐28.3)	.111^c^
	(n = 72)	(n = 37)	(n = 35)	

Abbreviations: CCND2, cyclin D2; OLIG2, oligodendrocyte lineage transcription factor 2; GBM, glioblastoma; IDH, isocitrate dehydrogenase; NA, not applicable; NOS, not otherwise specified; RT, radiotherapy; RT‐TMZ, radio‐chemotherapy with temozolomide.

a *t* Test.

b Chi‐squared.

c Wilcoxon test.

### 2.2 IHC and semi‐quantitative analysis

Standard IHC was applied to 5‐μm thick sections to display OLIG2 and CCND2 expression using respectively a specific antibody provided by Chemicon‐Millipore (ab9610, dilution 1:500) and ProteintechGroup Inc (Rabbit polyclonal, 10934‐1‐AP, dilution 1:150). Immunohistochemistry was performed on the BONDMAX. Briefly, as previously described,^13^ the immunohistochemical expression was visualized by means of streptavidin‐biotin‐peroxidase complex kit reagents (BioGenex) with diaminobenzidine/H_2_O_2_ as chromogenic substrate. Finally, the sections were counterstained with hematoxylin. IHC method was chosen instead of RNA sequencing analysis to allow precise in situ localization of the protein expression within the analyzed tissue.^14^ Semi‐quantitative analysis was performed by two independent observers (CB and ALT). The staining was assessed by means of two features: staining intensity (absent, low, moderate or strong) and labeling index (0: no staining; low nuclear expression <30% and high nuclear expression ≥30%). The labeling index was determined by random selection of five fields of representative tumor blocks at 40× power magnification. For the few cases where there was a discrepancy between the two scores obtained, a third observer (PD) assessed the final index.

### 2.3 Statistics

Statistical analyses were performed using Stata 14. The normal distribution of the data was verified using histograms, boxplots, and quantile‐quantile plots, and the equality of variances was checked using the Levene's test.

Since our study included a combination of GBM patients treated with surgical resection plus RT alone or RT‐TMZ, we chose 12 months as cut‐off for early mortality because it is the shortest median survival obtained in the major randomized phase III trial by Stupp et al.^3^ Thus, we divided our sample (n = 72) into a group with late mortality (≥12 months, from the date of the initial surgery) (n = 51) and a group with early mortality (<12 months, from the date of the initial surgery) (n = 21).

Categorical data were described with percentages and numbers, and continuous data were described with means and SD or median and interquartile range. Normally distributed variables were analysed with a *t* test. A Wilcoxon test or chi‐squared test was used on asymmetric distributed or dichotomous variables.

Univariate and multivariate binary logistic regression models were used to study the effects of risk factors on the occurrence of early mortality. Risk factor variables included number of lesions (categorical: unique, multiple), type of initial surgery (categorical: total, subtotal), type of adjuvant treatment (categorical: radiotherapy alone, radio‐chemotherapy), type of surgery at recurrence (categorical: total, subtotal), age (categorical: <50 years, ≥50 years), time to recurrence (categorical: <6 months, ≥6 months), nuclear expression of CCND2 before at initial surgery (categorical: <30%, ≥30%), nuclear expression of CCND2 after adjuvant treatment (categorical: <30%, ≥30%), nuclear expression of OLIG2 at initial surgery (categorical: <30%, ≥30%), nuclear expression of OLIG2 after adjuvant treatment (categorical: <30%, ≥30%), and as binary variables for gender and preoperative corticosteroids. Cut‐off values of 30% for the protein expression of CCND2 and OLIG2 were chosen because these expression levels were associated with the best sensitivity and specificity for the prediction of early mortality in our GBM cohort.

The automatic selection of risk factors in the model was performed by a stepwise backward method with an entry threshold of 0.05 and an exit threshold of 0.1. The adequacy of the model was verified by the Hosmer‐Lemeshow test, and the specificity of model was verified by the Link test. The other conditions of application of the multivariate logistic regression (number of subjects by risk factors, outliers, and collinearity between risk factors) were also verified. A *P*‐value of less than .05 was considered significant.

## 3 RESULTS

### 3.1 Semi‐quantitative analysis of OLIG2 and CCND2 protein expression

**Figure 1:**
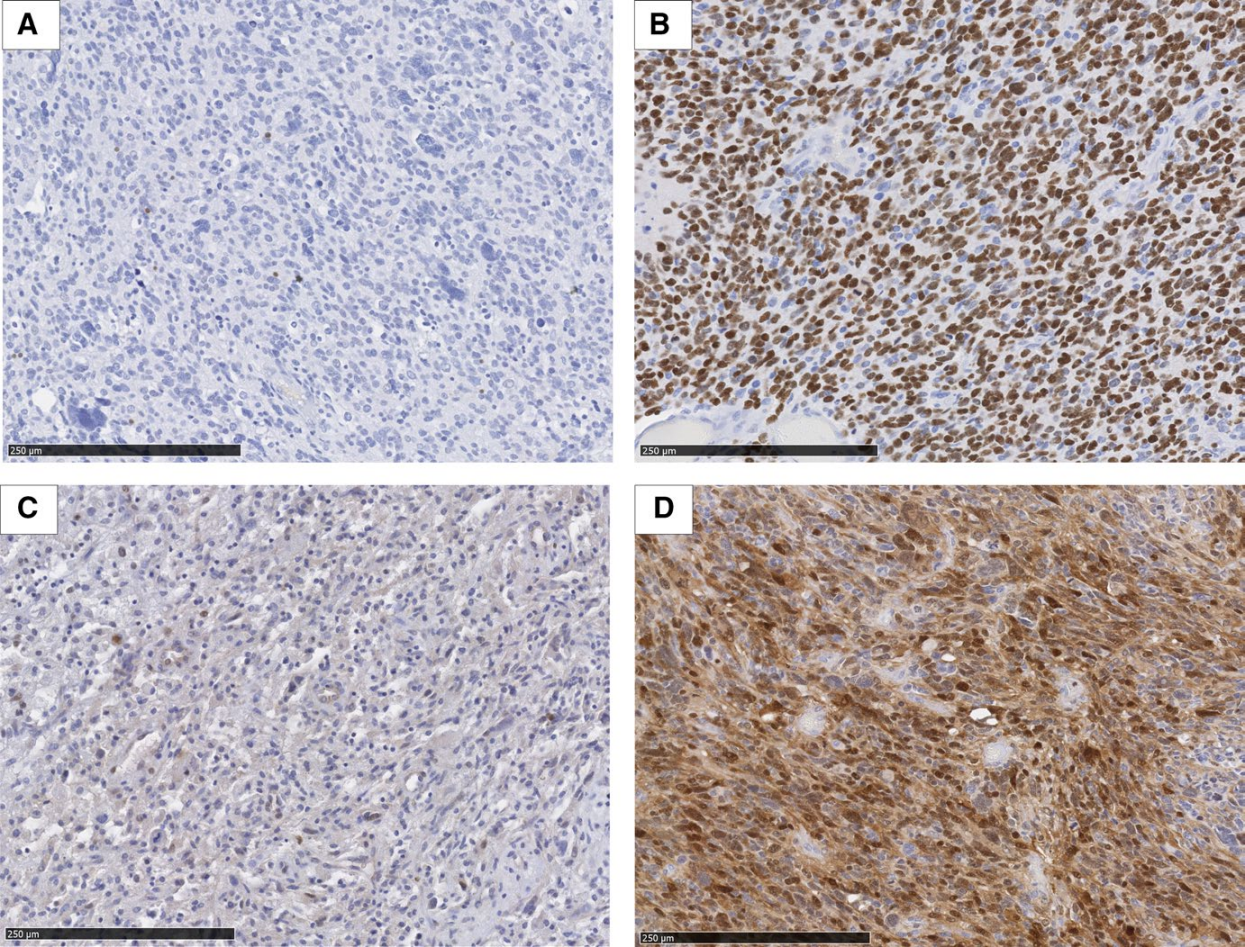
Cyclin D2 (CCND2) and oligodendrocyte lineage transcription factor 2 (OLIG2) expression profile in glioblastoma. CCND2 and OLIG2 present a nuclear and a cytoplasmic expression in glioblastoma. A, Low nuclear expression of OLIG2 (<30% of tumor cells nuclei stained). B, High nuclear expression of OLIG2 (≥30% of tumor cells nuclei stained). C, Low nuclear expression of CCND2 (<30% of tumor cells nuclei stained). D, High nuclear expression of CCND2 (≥30% of tumor cells nuclei stained)

Olig2 expression was predominantly nuclear and cytoplasmic in fewer cases. Almost all cases were positive, only one case presented negative paired tumor samples and two cases became negative at second surgery. The staining intensity was moderate‐to‐strong in all positive tumors. Ccnd2 expression was nuclear and cytoplasmic. All cases were positive for nuclear staining at initial surgery; only one case became negative at second surgery. The nuclear staining intensity was moderate to strong in all positive tumors. (Figure 1).

### 3.2 Sample description according to adjuvant treatment received

Compared to initial samples, Ccnd2 and Olig2 expressions were significantly decreased in GBM recurrences. The decrease in expression observed did not differ significantly according to the treatment used. The RT alone group relapsed faster and had to be re‐operated more quickly than the RT‐TMZ group. There were no significant differences for other parameters (Table 1).

### 3.3 Sample description according to mortality

Subtotal second surgery for recurrence, time to recurrence <6 months, Ccnd2 nuclear expression at initial surgery ≥30%, and Olig2 nuclear expression <30% at second surgery after RT alone and RT‐TMZ were more frequent in patients with early mortality. Patients with early mortality presented shorter time to recurrence and lower Olig2 expression after adjuvant treatment than the patients with late mortality. There were no significant differences for other parameters (Table 2).

**Table 2:** Sample description according to mortality (n = 72)

Variables	Whole cohort	Categories	%	Late mortality ≥12 mo (n = 51)	Early mortality <12 mo (n = 21)	*P*‐value Chi‐squared
Gender		Male (n = 45)	62.50	62.75% (n = 32)	61.90% (n = 13)	.947
		Female (n = 27)	37.50	37.25% (n = 19)	38.10% (n = 8)	
Number of lesions		Unique (n = 63)	87.50	87.27% (n = 44)	90.48% (n = 19)	.624
		Multiple (n = 9)	12.50	13.73% (n = 7)	9.52% (n = 2)	
Preoperative corticosteroids		No (n = 14)	19.44	21.57% (n = 11)	14.29% (n = 3)	.478
		Yes (n = 58)	80.56	78.43% (n = 40)	85.71% (n = 18)	
Type of initial surgery		Total (n = 43)	59.72	66.67% (n = 34)	42.86% (n = 9)	.061
		Subtotal (n = 29)	40.28	33.33% (n = 17)	57.14% (n = 12)	
Adjuvant treatment		RT alone (n = 37)	51.39	47.06% (n = 24)	61.90% (n = 13)	.252
		RT‐TMZ (n = 35)	48.61	52.94% (n = 27)	38.10% (n = 8)	
Type of surgery at recurrence		Total (n = 31)	43.06	50.98% (n = 26)	23.81% (n = 5)	.034
		Subtotal (n = 41)	56.94	49.02% (n = 25)	76.19% (n = 16)	

	Mean ± SD					*t* Test
Age at diagnosis (y)	54.23 ± 11.63			54.23 ± 11.63	56.39 ± 10.85	.469
		<50 (n = 20)	27.78	25.49% (n = 13)	33.33% (n = 7)	.499^a^
		≥50 (n = 52)	72.22	74.51% (n = 38)	66.67% (n = 14)	

	Median (P25‐P75)					Wilcoxon test
Overall survival (mo)	15.69 (11.63‐23.14)			19.40 (15.19‐25.90)	9.94 (8.43‐11.13)	<.001
Time to recurrence (mo)	6.90 (4.54‐10.90)			9.03 (6.00‐13.43)	4.33 (2.97‐5.00)	<.001
		>6 (n = 42)	58.33		19.05% (n = 4)	<.001^a^
		≤6 (n = 30)	41.67	74.51% (n = 38)	80.95% (n = 17)	
				25.49% (n = 13)		
CCND2 nuclear expression (%) at T0	30 (10‐50)			25 (10‐45)	40 (30‐50)	.086
		<30 (n = 30)	41.67	50.98% (n = 26)	19.05% (n = 4)	.012^a^
		≥30 (n = 42)	58.33	49.02% (n = 25)	80.95% (n = 17)	
CCND2 nuclear expression (%) at T1	12.5 (5‐30)			15 (5‐35)	10 (5‐25)	.528
		<30 (n = 53)	73.71	70.59% (n = 36)	80.95% (n = 17)	.364^a^
		≥30 (n = 19)	26.39	29.41% (n = 15)	19.05% (n = 4)	
OLIG2 nuclear expression (%) at T0	50 (20‐60)			50 (25‐70)	30 (20‐50)	.119
		<30 (n = 20)	27.78	25.49% (n = 13)	33.33% (n = 7)	.499^a^
		≥30 (n = 52)	72.22	74.51% (n = 38)	66.67% (n = 14)	
OLIG2 nuclear expression (%) at T1	30 (10‐50)			30 (20‐50)	20 (10‐25)	.022
		<30 (n = 35)	48.61	37.25% (n = 19)	76.19% (n = 16)	.003^a^
		≥30 (n = 37)	51.39	62.75% (n = 32)	23.81% (n = 5)	

Abbreviations: CCND2, cyclin D2; OLIG2, oligodendrocyte lineage transcription factor 2; RT, radiotherapy; RT‐TMZ, radio‐chemotherapy with temozolomide; SD, standard deviation; T0, at initial surgery; T1, at recurrence after adjuvant treatment.

a Chi‐squared.

### 3.4 Univariate analysis and Multivariate analysis

Similar to the univariate analysis (Data S1), risk factors obtained by the method of automatic selection (stepwise backward) and significantly associated with an increased risk of early mortality were: subtotal surgery at recurrence, time to recurrence <6 months, Ccnd2 nuclear expression at initial surgery ≥30%, and Olig2 nuclear expression <30% at second surgery after RT alone and RT‐TMZ (Table 3).

**Table 3:** Summary of logistic regression for screening variables predicting early mortality in glioblastoma patients (n = 72)

Variables	Adjusted OR (CI 95%)	*P*‐value
Time to recurrence (mo)		.002
>6 (n = 42)	1	
≤6 (n = 30)	16.85 (2.78‐102.2)	
Type of surgery at recurrence		.006
Total (n = 31)	1	
Subtotal (n = 41)	16.54 (2.20‐124.23)	
CCND2 nuclear expression (%) at T0		.012
<30 (n = 30)	1	
≥30 (n = 42)	14.33 (1.80‐114.27)	
OLIG2 nuclear expression (%) at T1		.023
<30 (n = 35)	1	
≥30 (n = 37)	0.14 (0.03‐0.76)	

Note

Not included in the model because not significant: gender, age, number of lesions, preoperative corticosteroids, initial lesion surgery, adjuvant treatment, CCND2 nuclear expression after adjuvant treatment and OLIG2 expression at initial surgery.

Adequacy of model: Hosmer‐Lemeshow chi2 (*P* = .947).

Specificity of model: Linktest (linear component *P* = .001 and nonlinear component *P* = .899).

Abbreviations: CI, confidence interval; CCND2, cyclin D2; OLIG2, oligodendrocyte lineage transcription factor 2; OR, odd ratio; T0, at initial surgery; T1, at recurrence after adjuvant treatment.

### 3.5 Characteristics of the sample according to the evolution of Olig2 expression in individuals with initial Olig2 labeling index ≥30%

In individuals with initial nuclear Olig2 expression ≥30%, the decrease in Olig2 expression <30% at recurrence is associated with earlier relapse, shorter time between the two surgeries and poorer survival. There were no significant differences for other parameters (Table 4).

**Table 4:** Characteristics of the sample according to the post‐treatment evolution of OLIG2 expression in glioblastoma patients with initial OLIG2 labeling index ≥30% (n = 52)

	Whole sample	OLIG2 < 30% (n = 23)	OLIG 2 ≥ 30% (n = 29)	*P*‐value
Age at diagnosis	56.44 ± 11.13	55.64 ± 13.06	57.08 ± 9.53	.119^a^
Gender (male)	57.69%	52.17%	62.07%	.473^b^
Number of lesions (multiple)	9.62%	8.70%	10.34%	.841^b^
Preoperative corticosteroids (yes)	75%	73.91%	75.86%	.872^b^
Time to recurrence (mo)	6.74 (4.57‐10.65)	5.37 (4.20‐7.27)	8.67 (6.17‐13.43)	.007^c^
Time between surgery (mo)	7.96 (5.62‐11.79)	6.30 (4.72‐7.84)	10.03 (7.37‐13.37)	.001^c^
Type of surgery at recurrence (subtotal)	51.92%	47.83%	55.17%	.598^b^
Overall survival (mo)	15.52 (11.84‐23.14)	12.57 (9.90‐15.37)	19.45 (14.83‐27.38)	<.001^c^

Abbreviations: OLIG2, oligodendrocyte lineage transcription factor 2.

a *t* Test.

b Chi‐squared.

c Wilcoxon test.

## 4 DISCUSSION

Glioma stem cells are critical cells implicated into GBM recurrence and radio(chemo)resistance through multiple and not fully elucidated mechanisms.^15^ The GSCs need urgently to be targeted and eliminated to one day offer a chance for cure for GBM patients. Unfortunately, the GSC population is highly heterogeneous even within a same tumor and shows phenotypic plasticity abilities between subpopulations.^7^, ^16^, ^17^, ^18^, ^19^, ^20^, ^21^ Two clinically relevant molecular subtypes signatures of GBM have consistently been highlighted between studies based on genetic and RNA expression profiles, namely the proneural (PN) and the mesenchymal (MES) which is associated with the worst prognosis.^22^, ^23^, ^24^, ^25^, ^26^ These two subtypes have also been identified for GSCs and seemed to be mutually exclusive.^7^, ^16^, ^21^, ^27^ Some data suggest that all GSC subtypes evolve from the PN phenotype, which is closely linked to OLIG2.^20^, ^28^ Olig2 is a bHLH transcriptional repressor protein that plays a critical role during the central nervous system development by maintaining glial progenitor cells in a competent proliferation state and allowing their specification.^29^, ^30^, ^31^, ^32^, ^33^, ^34^, ^35^ Olig2 is ubiquitously expressed in gliomas, irrespectively of grade, in various extents and is implicated in the gliomagenesis.^36^, ^37^, ^38^, ^39^, ^40^, ^41^ Previous studies have recognized Olig2 as a potential GSCs biomarker and it is one of the four transcription factors (with Sox2, Pouf3f2 and Sall2) that are sufficient to reprogram differentiated cells into GSCs.^42^, ^43^, ^44^ In the same way, we have recently highlighted the fact that Olig2 seems to be the best GSCs biomarker when comparing differentially expressed genes between differentiated and GSCs enriched cultures. Furthermore, Olig2 can easily be studied by IHC as we previously demonstrated in a small cohort of GBM.^12^ Olig2 is also a surrogate marker of the PN subtype of GSC, used instead of CD133 as this key PN marker can be studied by FACS but not by IHC.^17^, ^21^, ^26^ The PN phenotype seems to be predominant at the tumor edge based on gene expression profile.^17^, ^21^, ^45^, ^46^ However, in the present study, the nuclear labeling of Olig2 in the tumor core of initial surgery samples was found in majority of the cases (n = 71/72) with high nuclear expression (≥30%, n = 52/72). PN phenotype is sometimes associated with a better survival than MES subtype as illustrated by Pinel et al where high level of Olig2 expression tended to be associated with a better overall survival on TMA samples of 80 GBM.^47^ In our cohort, initial nuclear expression of Olig2 ≥30% is not associated with a significant improvement in survival. For patients with initial Olig2 expression ≥30%, a decrease in Olig2 expression (<30%) at second surgery is significantly associated with a shorter time to recurrence (5.37 months vs 8.67 months, *P* = .007) and poorer overall survival (12.57 months vs 19.45 months, *P* < .001). This could be explained by the fact that initial PN phenotype shifts into a more aggressive MES subtype induced by treatments, whether RT alone or RT‐TMZ.^22^ There is now more evidence that GBM and particularly GSCs exhibit phenotypic plasticity, particularly following (chemo‐)RT.^16^, ^18^, ^19^, ^21^, ^48^ Moreover, the maintenance of the oligodendrocyte precursor cells signature associated with the PN phenotype is linked to Olig2 expression.^43^, ^49^ Preclinical data showed that once OLIG2 was knocked down in PN GSCs lines, an upregulation of CD44 and others markers associated with MES phenotype was observed.^50^ The mechanisms behind this transition are not fully known but a beginning of response was described by Minata et al^21^ Following RT an upregulation of CD109, another MES marker, happens rapidly in PN GSCs (within 24 hours) with at the same time a reduction of CD133+ and Olig2+ GSCs. Radiation therapy induces the activation of nuclear factor κB through ATM activation by RT‐mediated DNA damage^51^, ^52^, ^53^, ^54^ resulting in the upregulation of CD109. The treatment‐naive or RT‐induced CD109+ GSCs are both highly tumorigenic and radioresistant.^21^ Other studies showed that PN to MES transition is also associated with global multidrug resistance.^16^, ^17^, ^44^, ^55^ All these data could therefore explain the poor prognosis associated with the decrease in Olig2 expression that we showed in GBM after adjuvant treatments. However, this shift appears to be not systematic after RT(‐TMZ) and further studies should investigate why and how PN‐to‐MES transition could be avoided.

We also studied another potential GCS marker, Ccnd2. The three D‐type cyclins (Cyclin D1, D2, and D3) are key checkpoint regulators of the mammalian cell cycle. Under the control of distinct intracellular pathway, they promote transition from G1 to S phase through activation of the cyclin‐dependent kinase Cdk4/6, phosphorylation of retinoblastoma suppressor protein (pRB), and suppression of pRB inhibitory function on E2F transcription factors ending in cell proliferation.^56^, ^57^ Cyclin D2 is necessary for normal gonadal cell proliferation and is crucial for neurogenesis.^58^, ^59^ Cyclin D2 is the predominant cyclin D in the human subventricular zone allowing expansion of the cortical intermediate progenitor cell population at embryonic stage and neurospheres of expanded neuronal precursors of adult hippocampi express only Ccnd2.^59^, ^60^ Cyclin D2 is rarely expressed in normal brain or low grade glioma and is significantly upregulated in GBM.^61^, ^62^, ^63^ Regarding GSCs, we have previously shown that CCND2 is part of the most differentially expressed genes between differentiated and GSC enriched cultures from similar DNA chip microarray platforms.^12^ In the same way, Koyama‐Nasu et al^63^ demonstrated by immunoblotting analysis and RT‐PCR that Ccnd2 is abundantly expressed only in undifferentiated GBM cell lines, which was not the case for Cyclin D1 and D3. Using siRNA, solely the knockdown of CCND2 resulted in a significant increase of G1 arrest of GSCs. Furthermore, Ccnd2 seems to have a critical role in the tumorigenicity as mice transplanted with GSCs in which Ccnd2 expression was repressed survived significantly longer than those maintaining an expression of Ccnd2.^63^ In the present study, we identified Ccnd2 nuclear expression at initial surgery ≥30% to be significantly associated with early mortality (<12 months), confirming the prognostic value of this GSCs marker in human. At recurrence, Ccnd2 expression is reduced and has no longer a prognostic impact. Therefore, targeting CCND2 before adjuvant RT‐TMZ could be a promising way to explore for GBM therapy.

Multivariate analysis and logistic regression also highlighted the fact that a short time to recurrence (≤6 months) and a subtotal surgery at recurrence are variables predicting early mortality (<12 months). It should be noted that subtotal initial surgery was not identified as a predictive variable as classically described but our cohort only included patients who presented a local recurrence and who could be re‐operated.^64^, ^65^, ^66^, ^67^ It may be therefore logically conceivable that we found only a predominant impact on survival for the type of surgery at recurrence (subtotal or not).

### 4.1 Limitations

Our study presents limitations given that the molecular data (MGMT methylation and IDH mutation status) are missing in older cases as these analyses were not yet implemented routinely at that time. Additional molecular sequencing could not be performed due to the retrospective nature of our study. Therefore, these data could not be included in our analyses. Furthermore, our study included a relatively small cohort of 72 patients with paired samples from a single center. However due to the low frequency of GBM patients eligible for repeat surgery,^68^ our paired cohort is to our knowledge one of the larger described in the literature.^69^, ^70^, ^71^, ^72^, ^73^, ^74^ Finally, we studied only the marker expression on the tumor core. In future studies, it might be interesting to analyse the expression of OLIG2 and CCND2 also at the invasive tumor edge and to perform validation of our biomarkers with different techniques such as RNA sequencing.

## 5 CONCLUSIONS

To our knowledge, we are the first to demonstrate on a paired cohort of human GBM that:

- A comparative IHC of OLIG2 realized at initial and recurrence surgery has prognostic value. The patients for whom nuclear expression of OLIG2 becomes low (<30%) after adjuvant treatments have a significantly shorter time to recurrence and survival reflecting most probably a PN to MES transition of the GSCs population.
- At initial surgery, high nuclear expression (≥30%) of CCND2, a G1/S regulator specific of GSCs, has prognostic value and is associated with early mortality (<12 months).

In the future, prospective studies should be conducted with GBM patient to validate the risk factors for early mortality highlighted in our study and explore the relationships of OLIG2 and CCND2 stem cell markers with the molecular status of GBM.

## CONFLICT OF INTEREST

None declared.

## AUTHOR CONTRIBUTIONS

CB contributed to acquisition, analysis and interpretation of data, and drafted the manuscript; ALT contributed to acquisition, analysis, and interpretation of data; MH contributed to statistical analysis and interpretation of data; DVG: contributed to interpretation of data; PD contributed to acquisition, analysis, and interpretation of data, conceptualization, and supervision of the study. All the authors reviewed and edited the manuscript, gave critical input on interpretation of results, and gave the final approval for publication.

## Data Availability

The data that support the findings of this study are available from the corresponding author upon reasonable request. The data are not publicly available due to privacy or ethical restrictions.
