# Supplementary material for: Prognostic impact of glioblastoma stem cell markers OLIG2 and CCND2
Source: Cancer Med. 2019 Sep 30;9(3):1069–78. doi: 10.1002/cam4.2592 (PMC6997071; doi:10.1002/cam4.2592)
Supplement: Supplementary file 1 [file CAM4-9-1069-s001.docx]

**Supplemental Table S1: Univariate analysis (n=72)**

| Variables | Categories | Group  Late Mortality  ≥12 months  (n=51) | Group  Early Mortality  <12 months  (n=21) | OR (CI 95%) | P-value |
| --- | --- | --- | --- | --- | --- |
| Gender | Male (n=45)  Female (n=27) | 71.11% (n=32)  70.37% (n=19) | 28.89% (n=13)  29.63% (n=8) | 0.96 (0.34 to 2.75)  1 | 0.947 |
| Age (years) | <50 (n=20)  ≥50 (n=52) | 65.00% (n=13)  73.08% (n=38) | 35.00% (n=7)  26.92% (n=14) | 1  0.68 (0.23 to 2.06) | 0.501 |
| Number of lesions | Unique (n=63)  Multiple (n=9) | 69.84% (n=44)  77.78% (n=7) | 30.16% (n=19)  22.22% (n=2) | 1  0.66 (0.13 to 3.48) | 0.626 |
| Preoperative corticosteroids | No (n=14)  Yes (n=58) | 78.57% (n=11)  68.97% (n=40) | 21.43% (n=3)  31.03% (n=18) | 1  1.65 (0.41 to 6.64) | 0.481 |
| Initial lesion surgery | Total (n=43)  Subtotal (n=29) | 79.07% (n=34)  58.62% (n=17) | 20.93% (n=9)  41.38% (n=12) | 1  2.67 (0.94 to 7.56) | 0.065 |
| Adjuvant treatment | Radiotherapy alone (n=37)  Radio-chemotherapy (n=35) | 64.86% (n=24)  77.14% (n=27) | 35.14%(n=13)  22.86% (n=8) | 1  0.55 (0.19 to 1.54) | 0.255 |
| Time to recurrence (months) | >6 (n=42)  ≤6 (n=30) | 90.48% (n=38)  43.33% (n=13) | 9.52% (n=4)  56.67% (n=17) | 1  12.42 (3.53 to 43.72) | <0.001 |
| Surgery recurrence | Total (n=31)  Subtotal (n=41) | 83.87% (n=26)  60.98% (n=25) | 16.13% (n=5)  39.02% (n=16) | 1  3.33 (1.06 to 10.45) | 0.039 |
| CCND2 nuclear expression (%) -  At initial surgery | <30 (n=30)  ≥30 (n=42) | 86.67% (n=26)  59.52% (n=25) | 13.33% (n=4)  40.48% (n=17) | 1  4.42 (1.31 to 14.97) | 0.017 |
| CCND2 nuclear expression (%) -  At recurrence | <30 (n=53)  ≥30 (n=19) | 67.92% (n=36)  78.95% (n=15) | 32.08% (n=17)  21.05% (n=4) | 1  0.56 (0.16 to 1.96) | 0.368 |
| OLIG2 nuclear expression (%) -  At initial surgery | <30 (n=20)  ≥30 (n=52) | 65.00% (n=13)  73.08% (n=38) | 35.00% (n=7)  26.92% (n=14) | 1  0.68 (0.23 to 2.06) | 0.501 |
| OLIG2 nuclear expression (%) -  At recurrence | <30 (n=35)  ≥30 (n=37) | 54.29% (n=19)  86.49% (n=32) | 45.71% (n=16)  13.51% (n=5) | 1  0.19 (0.06 to 0.59) | 0.004 |
|  |  |  |  |  |  |

OR = Odd Ratio, CI = **Confidence Interval**
